# Xiphodynia Caused by a Large Xiphoid Process

**DOI:** 10.7759/cureus.44516

**Published:** 2023-09-01

**Authors:** Tomoya Tsuchida, Kosuke Ishizuka, Yoshiyuki Ohira

**Affiliations:** 1 Internal Medicine, St. Marianna University School of Medicine, Kanagawa, JPN; 2 General Medicine, Yokohama City University School of Medicine, Yokohama, JPN; 3 Internal Medicine, St. Marianna University School of Medicine, Kawasaki, JPN

**Keywords:** differential diagnosis, risk factor, epigastric pain, large xiphoid process, xiphodynia

## Abstract

We present the case of a 72-year-old man with a three-month history of epigastric pain. A physical examination revealed a tender, hard mass around the epigastric area. Enhanced CT showed no chest or abdominal abnormalities, except for a large xiphoid process. The diagnosis was xiphodynia caused by a large xiphoid process. Xyphoidynia should be considered a differential diagnosis for epigastric pain.

## Introduction

The differential diagnosis for epigastric pain varies as it encompasses conditions including cardiac diseases and abdominal diseases such as gastroesophageal reflux disease (GERD) and Helicobacter pylori-related ulcers [1]. However, xiphodynia should also be considered as a differential diagnosis. The risk factors for xiphodynia include occupations involving heavy lifting, childbirth, and primary conditions such as GERD, coronary artery disease, and trauma. Other pathophysiologies such as osteoarthritis (OA), rheumatoid arthritis (RA), ankylosing spondylitis, Reiter’s triad, and psoriatic arthritis should be considered as differential diagnoses of epigastric pain since these may be associated with xiphodynia [1-4]. Despite the numerous risk factors associated with xiphodynia, few reports have focused on its size. In this study, we present a case of xiphodynia caused by a large xiphoid process.

## Case presentation

**Figure 1:**
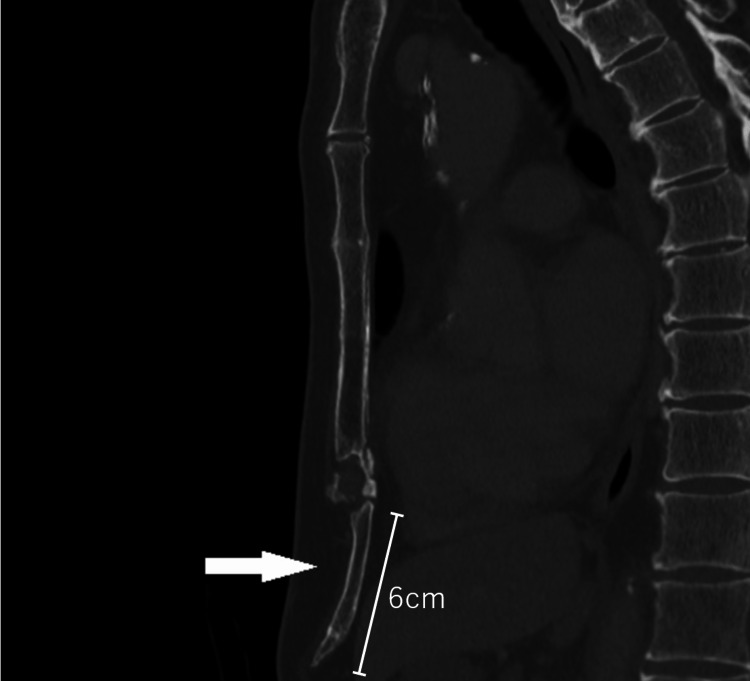
Large xiphoid process (white arrow) seen on CT

A 72-year-old Japanese man presented with chronic epigastric, not-so-severe, dull pain and nausea that had persisted for the previous three months. His medical history revealed hypertension, for which he was taking medication (losartan 50 mg/day). The patient was of medium build and had no history of chest or abdominal surgery. He retired from work seven years ago; however, he reported undertaking do-it-yourself (DIY) projects as a hobby. During physical examination, a tender, hard mass was detected around the epigastric area. A blood examination, electrocardiography, abdominal ultrasound examination, and gastroscopy detected no abnormalities. Enhanced CT revealed no chest or abdominal findings except for a large xiphoid process, measuring 6 cm (Figure 1). Based on this evidence, the patient was diagnosed with xiphodynia. After two weeks of treatment with non-steroidal anti-inflammatory drugs (NSAIDs), his symptoms resolved.

## Discussion

The epidemiological characteristics of xiphodynia remain unclear. Joint damage, potential subluxation, and other diseases such as OA and RA may be caused by xiphodynia [1]. Symptoms may include chest or epigastric pain, nausea, vomiting, and diarrhea, with radiating pain to the back, neck, shoulders, arms, and chest wall [3]. The diagnosis of xiphodynia relies on the reproduction of the patient's symptoms, either fully or partially, through moderate pressure on the xiphoid process [3]. However, serious conditions such as angina pectoris, myocardial infarction, pericarditis, gastric disease, and biliary disease should be ruled out for the diagnosis of xiphodynia [2,5].

The primary treatment for xiphodynia is conservative, involving NSAID treatment. If no improvement is observed, several local xylocaine or steroid injections may be necessary. Yet, if no improvement is observed, surgery may be effective [1]. Olivencia reported 40 patients who underwent xiphoidectomy for xiphodynia refractory to conservative treatment and showed predominant symptomatic improvement [6]. Addressing the underlying diseases that can cause xiphoidalgia is essential before treating the condition; however, studies comparing and evaluating the effectiveness of treatments for xiphoidalgia are limited, and standardized approaches are yet to be established [1].

Occupations involving heavy lifting, childbirth, trauma to the lower chest or abdomen, and anatomical variation of the xiphisternal angle are thought to be risk factors [1,7,8]. Stooping forward, bending the torso, and overeating are factors that can worsen the symptoms [1]. The xiphoid process is the most variable sternal element [9] and it is usually 2.77±1.5 cm in length [10]. However, in this case, the length was approximately 6 cm. A long xiphoid process may be a risk factor for xiphodynia, as evidenced by a 19-case series wherein more than half of the cases had a xiphoid process longer than 5 cm [11]. Enomoto et al. showed that xiphodynia can be caused by postoperative elongation of the xiphoid process, and Trang et al. reported a case of xiphodynia caused by an ossified large xiphoid process after chest compressions [12,13]. However, the relationship between the size of the xiphoid process and pain remains unknown. Additionally, minor trauma to the anterior chest wall can also cause xiphodynia [14]. In the present case, there was no obvious traumatic episode, although minor pressure on the large xiphoid process during the patient’s DIY projects may have been a causative factor.

## Conclusions

Herein, we describe a case of xiphodynia that may have originated from a large xiphoid process in a patient without any precipitating factor or known underlying disease for this process. Our case highlights that xiphodynia should be considered a differential diagnosis after excluding other more common pathologies in cases of induration of the xiphoid process with tenderness. A large-sized xiphoid process possibly constitutes a risk factor for xiphodynia.
